# The Coupling Coordination Evolutionary Analysis of Tourism-Ecological Environment-Public Service for the Yellow River Basin of China

**DOI:** 10.3390/ijerph19159315

**Published:** 2022-07-29

**Authors:** Zhenhui Huang, Wei Wei, Ying Han, Shuangying Ding, Ke Tang

**Affiliations:** 1 Center for Energy Environment & Economy Research, School of Management, Zhengzhou University, Zhengzhou 450001, China; 202022024010545@gs.zzu.edu.cn (Z.H.); weiwei123@zzu.edu.cn (W.W.); hanyy925@gs.zzu.edu.cn (Y.H.); dsy929890127@gs.zzu.edu.cn (S.D.); 2 Research Center for Economic Development and Environment of the Yellow River Basin, Zhengzhou University, Zhengzhou 450001, China; 3 Yellow River Institute for Ecological Protection & Regionally Coordinated Development, Zhengzhou University, Zhengzhou 450001, China

**Keywords:** tourism, ecological environment, public service, Yellow River Basin, coupling coordination degree

## Abstract

In the context of the global pandemic, the development of tourism in the Yellow River Basin is constrained by the dual mechanisms of the decline in the quality of public service and the deterioration of the ecological environment. In order to promote the high-quality development of the ecological environment in the Yellow River Basin, this paper studies the coordinated development of tourism, the ecological environment and public service in the Yellow River Basin by treating tourism, the ecological environment and public service as a whole. Based on the coupling coordination function GM (1,1) grey prediction method and PVAR model, we discuss the characteristics of spatio-temporal differences, evolutionary trends and the interaction mechanism of the coupling coordination degree (CCD) of tourism-ecological environment-public service in nine provinces along the Yellow River Basin in China from 2008 to 2019. The results show that tourism and public service in the Yellow River Basin are closely related, and the protection of the ecological environment and tourism development are not contradictory. In terms of time, the overall trend is stable and upward from the perspective of the CCD of the three systems; in terms of space, Henan, Shandong, and Sichuan provinces have a relatively high level of CCD. While Qinghai, Gansu, Ningxia, Inner Mongolia, Shaanxi and Shanxi provinces have a lower level of CCD, which shows an upward trend from upstream to downstream in the space. The evolutionary trend of the CCD of the three systems in the basin will be upward in all provinces except for the Shandong province in the next five years. Tourism can promote both the ecological environment and public service from the perspective of the mutual influence mechanism.

## 1. Introduction

Since the outbreak of the global epidemic of COVID-19, some scholars have paid much more attention to public health issues [1,2]. At the same time, the improper development of tourism has caused the deterioration of the regional ecological environment, which has also affected public service and restricted the regional economy and the harmonious development of society [3]. As the mother river of China, the Yellow River Basin is an important economic zone and an energy-intensive area for energy, oil and natural gas in China, as well as an important ecological protection barrier in northern China. However, due to the acceleration of urbanization and the gradual increase in per capita resource consumption, the ecological environment of the Yellow River Basin is deteriorating [4,5]. Therefore, it is necessary to take a series of effective measures to improve the ecological conservation and restoration capabilities and restore the ecological barrier of the Yellow River Basin. In addition, the serious soil erosion in the upper reaches of the Yellow River Basin has degraded the ecological environment in the basin continuously and caused other problems. Improving the quality of public service in the Yellow River Basin can be a big challenge. Actually, increasing investment in public service infrastructure can improve the quality of urban and rural public service facilities to some extent, narrow the regional gap, promote the equalization of urban and rural infrastructure systems and public service systems, and further ensure food production and national security in the Yellow River Basin. To sum up, tourism, the ecological environment and public service in the Yellow River Basin are interrelated. To be specific, improving the quality of the ecological environment can ensure the health of residents and improve the level of regional public service infrastructure, and promote the development of tourism in the Yellow River Basin. In turn, the health of residents provides a guarantee for tourism development, which promotes regional public service infrastructure, so as to further promote the restoration and protection of the ecological environment.

Under the background of China’s proposed ecological protection and high-quality strategy in the Yellow River Basin, this paper finds that the three systems of tourism, ecological environment and public service in the Yellow River Basin are complex and interrelated. According to the World Trade Report (UNWTO 2018), tourism is already the most polluting industry in the world. Some scholars believe that reducing CO_2_ emissions can improve the quality of the regional ecological environment and effectively alleviate the contradiction between tourism and the ecological environment [6]. Some scholars believe that the coordinated development of tourism and public service is conducive to the high-quality development of the regional economy [7]. Therefore, the relative research on the coordinated relationship between tourism, the ecological environment and sustainable development of public service has become a hot spot of common concern among academic circles and policymakers.

Current scholars mainly focus on two aspects such as tourism and the ecological environment [8], the ecological environment and public service [9], and tourism and public service [10] in the basin. However, less research has been conducted on the coordinated relationship between the merits and demerits of the ecological environment, the development of the tourism industry and the level of public service in the Yellow River Basin. To achieve the sustainable development of the tourism industry in the Yellow River Basin, it is necessary to improve the quality of public service and protect the ecological environment. According to the elements involved in the Outline of the Yellow River Basin’s Ecological Protection and High-quality Development Plan, tourism, ecological environment and public service are comprehensively selected as subsystems in this paper. Based on the coupling function and the CCD function, the coupling relationship between tourism-ecological environment-public service in the Yellow River Basin is explored in this paper. The GM (1,1) grey prediction method is adopted to predict the coordinated development of the three systems in the next five years, the Panel VAR (vector auto regression) model is used to explore the interaction among the three subsystems of tourism-ecological environment-public service in the basin, so as to provide a reference for the formulation of policies related to ecological protection and high-quality development in the Yellow River basin.

The major contributions of our paper can be summarized as follows:

From the perspective of the application, the policy recommendations provided in the paper can be helpful for policy design. In addition, scientific policies can improve the coordinated development level of tourism, ecological environment and public service in the Yellow River Basin, and promote the high-quality development of tourism in the Yellow River Basin, which is in line with our research purposes.

At the same time, the CCD model is used to explore the coordination relationship between tourism, the ecological environment and public service in the nine provinces of the Yellow River Basin, which breaks through the limitations of the coupling relationship between the two. Reasonable prediction of public service CCD will help to optimize the tourism industry structure, protect the ecological environment, improve infrastructure, and provide decision-making guidance in the future. The PVAR model is used to explore the mutual influence between tourism and public service in the basin, and we find that tourism, the ecological environment and public service have a unilateral promoting effect, and provide a reference for decision makers to adjust industrial development. It can also make up for the insufficiency of the current research methods on the interaction between subsystems in coordinated development and provide an auxiliary reference for other scholars. In the future, we will conduct a more detailed study of the prefecture-level cities along the Yellow River Basin. In addition, one-third of the weight is allocated to the three subsystems of tourism, ecological environment and public service in this study, the allocation mode can be more reasonable in the future study.

## 2. Literature Review

The Yellow River Basin is an important ecological barrier and important economic zone in China. The unique natural resource conditions, profound cultural heritage and good industrial foundation in the basin provide rich and diverse tourism resources along the Yellow River, which has laid a foundation for the high-quality development of tourism in the Yellow River Basin. Public service should be taken as the basis and ecological environment protection as the core of tourism development in the Yellow River Basin, so as to realize the high-quality and coordinated development of tourism, the ecological environment and public service in the Yellow River Basin. Therefore, it is of great practical significance to study the coupling relationship between tourism, the ecological environment and public service and grasp the dynamic evolution law of the three, so as to achieve the high-quality development of the tourism economy in the Yellow River Basin, improve the construction of the ecological environment protection system and improve tourism public service.

At present, the research on the relationship between tourism, the ecological environment and public service is still in the period of exploration. At this stage, scholars mainly focus on ecological environment protection [11], resources and environment [12], water resources utilization [13], sustainable development [14], human settlements [15], industrial structure [16,17], urban agglomeration construction [18,19], public service [20,21,22] urbanization [23], etc. In terms of tourism, Guan [24] used the coupling model to explore the complex coupling relationship between the tourism environment and tourism economy in Nanjing, China. The research found that the transportation environment and tourism benefits will be the key factors that need to be considered when making macro-decisions.. Chun [25] used the coupling model and GIS spatial analysis to analyze the correlation degree, coupling degree and coordination degree of ecological environment and urbanization in Chongqing, China, and put forward the sustainable development model of urban cultural tourism, ecology, industry and life service. In terms of ecological environment, Li [26] used the coupling model to explore the coordinated development of production life ecological space in the Yellow River Basin, which plays an important role in future regional planning and management. Tang [27] established the economic benefit ecological quality tourism CCD model for Heilongjiang province, China, whose results show that economic benefit and ecological quality have the greatest impact on the coupling system and are the key factors to be considered in macro decision-making. As for public service, Ming [28] used the empirical investigation to discuss the question that regional tourism destination managers should encourage tourists and local residents to participate in the formulation of public service standards, so as to effectively improve the local ecological environment and tourism quality. At the same time, the research methods also tend to be diversified. The main methods adopted are the data envelopment analysis method, the coupling coordination evaluation method, and the PSR model, and corresponding policy suggestions are put forward based on the model calculation results [29,30,31].

Coupling coordination was used in the field of physics in the early days, which mainly concentrated on the exploration of the close relationship between multiple subsystems. With the in-depth and excavation of scholars’ research, many scholars also applied coupling theory to tourism, ecological environment, etc. [32,33]. The coordinated development is also emphasized, Wang et al. used the coupling coordination theory to study the coordinated development level of low-carbon industries and tourism in Shenzhen and put forward relevant policy recommendations for its development into a low-carbon tourism city [34]. Even some scholars, Suk S et al., used the PSR model to couple and coordinate the tourism economy and the ecological environment in Nagasaki, Japan, revealing the dynamic trend of the coupled development of the tourism economy and the ecological environment [35].

With the help of the CCD function, the construction of the evaluation system and calculation provides a new direction for the development evaluation research of the tourism industry [36]. With the development and expansion of the tourism industry, the relationship between tourism, the ecological environment and public service is much closer, and the boundaries are blurred gradually. There are dynamic changes in the three systems, but few scholars have noticed the impact of the three systems on the development of the tourism industry. The development of the tourism industry is affected by many factors, such as economy, politics, culture, and environment, the indicators of which cannot be listed completely. However, the degree of synergy among the ecological environment, public service and tourism cannot be ignored, which is worth studying. Most of the existing research focuses on the influencing factors in the development of the tourism industry from two different perspectives in the three systems of ecological environment, public service and tourism, which is not conducive to a deep understanding of the relationship and interaction among the development of the tourism industry, the ecological environment and the public service system.

To sum up, improvements have been made in the research on the CCD of tourism-ecological environment-public service, which promotes the in-depth application of coupling coordination theory in tourism research. However, there are still some weaknesses. Firstly, the research on the interaction between subsystems does not go further, the theoretical discussion and empirical analysis of which are insufficient without unified research cognition and system construction. Secondly, most scholars at home and abroad study regional areas, and most of the areas are developed cities and countries. However, there are few studies on this special area of the Yellow River Basin. Thirdly, there are many studies on the coordination and mutual influence of tourism-ecological environment-public service, but few studies are on the coupling and coordinated development of tourism-ecological environment-public service, which makes it difficult to grasp the influencing factors in the development of the tourism industry in the Yellow River Basin from different perspectives. Therefore, in order to know more about the development status of the tourism industry from multiple perspectives, the CCD, the GM (1,1) grey prediction method and the PVAR model are used in this paper to discuss the tourism-ecological environment-public service CCD in the nine provinces of the Yellow River Basin from 2008 to 2019, so as to dig deeper into the relationship and interaction law between various systems.

## 3. Research Methods and Data Sources

### 3.1. Coupling Mechanism of Tourism-Ecological Environment-Public Service

Coupling means that two or more subsystems interact with each other [37]. CCD not only reflects the quality of the system level, but also reflects the harmonious relationship between the systems. Tourism-ecological environment-public service are important components of ecological protection and high-quality development in the Yellow River Basin. The coordinated development and trend of the three subsystems are bound to affect the ecological protection and high-quality development of the Yellow River Basin. (1) The development of tourism can promote the adjustment of tourism industrial structure and accelerate the transformation, upgrading and rationalization of regional industrial structure, so as to improve regional ecological efficiency. It is also an important driving force for ecological environment protection. At the same time, the high-quality development of tourism will reduce the consumption of resources and products in the region and is also conducive to the protection of the regional ecological environment. (2) Improving the quality of public service will optimize the quality of the ecological environment, which is also a necessary way to stimulate regional economic growth. Therefore, improving the quality of public service in the region is beneficial to regional economic growth, as well as to the optimization and development of the regional ecological environment. (3) Intensifying the protection of the regional ecological environment will improve the sense of happiness and belonging of regional residents. A suitable place for employment, residence and tourism can reduce the outflow of the regional labor force, thereby promoting the development of regional industries and the rationalization of the upgrading of the industrial structure.

To sum up, it can be seen that tourism-ecological environment-public service are coordinated with each other. Anyone in the poor system will affect the ecological protection and high-quality development of the Yellow River Basin. Therefore, it is of great practical significance to measure and analyze the CCD of tourism-ecological environment-public service in the Yellow River Basin.

### 3.2. Evaluation Index System and Data Resources

A comprehensive and complex system has been formed in tourism, the ecological environment and public service systems. The scale and benefit of tourism development, ecological balance and environmental quality, the improvement of public service infrastructure and the equalization of scientific research and education are all important factors affecting the regional high-quality development. To formulate a set of index systems that can comprehensively evaluate the development level of tourism-ecological environment-public service is the basic premise for analyzing the degree of regional coupling and coordination. Therefore, whether the construction of the evaluation index system is scientific and reasonable will greatly affect the accuracy of the evaluation results.

Based on existing theoretical achievements [38], and according to the principles of scientificity, systematization, and operability, three subsystems of tourism, ecological environment and public service are selected to measure the coordinated development of tourism in the Yellow River Basin. Tourism-ecological environment-public service is an open system with complexity, uncertainty and multi-level. To construct a comprehensive, systematic and scientific evaluation system is the premise of measuring the development level of the three systems. Drawing on the research results of existing scholars on the coordinated development evaluation of tourism, the ecological environment and public service [39], an index system is established, as shown in Table 1. However, there are still some problems. Firstly, the tourism market and tourism structure are taken into consideration when the original indicator measures the tourism subsystem. Based on the outline of the Yellow River development strategy, this paper re-evaluates the tourism subsystem from the two dimensions of tourism scale and tourism benefit. At the same time, the newly added indicators of the number of inbound tourists, domestic tourists, and tourism practitioners can reflect the scale of regional tourism development. Meanwhile, the indicators of per capita GDP and total social consumption and retail sales can be added to reflect the level of regional tourism industry benefits. Secondly, the status and governance of the ecological environment are emphasized in the original index, while the differences between the nine provinces in the Yellow River Basin are prominent. Furthermore, the ecological pollution in the Yellow River Basin is intensifying, but the restoration is slow. Therefore, on the basis of the original index system of the ecological environment, new indicators are added to reflect the situation. For example, the total amount of energy consumption, carbon dioxide and sewage discharge can reflect the intensity of damage to the regional environment. Moreover, wetland areas, forest areas and afforestation areas are added to reflect the improvement of regional ecological environment quality. Thirdly, the original indicator did not measure public service, but the biggest feature of tourism is to provide service. The level of tourism development can be seen from public service in the region, so public service is added as one of the subsystems of the evaluation system in this paper. Fourthly, some indicators are less representative or repeated, such as the operating income of tourism enterprises and the number of tourism employees. The two indicators have obvious positive correlations and have basically the same meaning, so the number of tourism employees is retained. Finally, under the guidance of the principles of scientificity, systematization, operability, and availability, three first-level indicators, six second-level indicators, and twenty-seven third-level indicators of ecotourism, ecological environment and public service are chosen as the final evaluation index system, as shown in Table 1.

The main sources of data in this article: China Forestry Statistical Yearbook, China Agricultural Statistical Yearbook, China Culture and Tourism Yearbook, and provincial statistical yearbooks and statistical bulletins of national economic and social development from 2009 to 2020, in which some missing data is compensated by adopting the method of linear interpolation.

### 3.3. Research Methods

#### 3.3.1. Entropy Method

Andria [40] believes that the advantage of the entropy method is that its availability allows ambiguous or vague statements, which can reduce the subjectivity in the evaluation process. Thus, the resulting index weights are made more objective, and effectively eliminate the impact of different data, so that a unified standard can be used to compare the index data of the evaluation objects over the years. Therefore, the range method is first adopted in this paper to standardize the index data. Then, the entropy method is adopted to weight the index of tourism, the ecological environment and public service. The specific calculation process is as follows:

(1) Data standardization. Let $y_{ij}$ be the dimensionless value of the j-th index and the i-th evaluation object, $x_{ij}$ be the original data and the j-th index of the i-th evaluation object, and n be the object to be evaluated. Then, different strategies of the forward index and reverse index under the same evaluation object are standardized by:
  (1) $$\mathrm{Positive}\;\mathrm{indicators}:y_{ij}=\frac{x_{ij}-\min(x_{ij})}{\max(x_{ij})-\min(x_{ij})}+0.001$$
  (2) $$\mathrm{Reverse}\;\mathrm{index}:y_{ij}=\frac{\max(x_{ij})-x_{ij}}{\max(x_{ij})-\min(x_{ij})}+0.001$$
(2) The entropy weight method is used to weight each index in the index system. The information entropy e *_j_* of the j-th index is calculated by,
  (3) $$e_{j}=-k\overset{n}{\underset{i=1}{\sum }}p_{ij}\ln p_{ij}(i=1,2,3\ldots m;j=1,2,3\ldots n)$$
  where $p_{ij}=y_{ij}/\overset{n}{\underset{i=1}{\sum }}y_{ij},k=1/\ln n$.
(3) Calculate the difference coefficient $g_{j}\;$ of the j-th index:
  (4) $$g_{j}=1-e_{j}$$
(4) Calculate the weight $w_{j}$ of the j-th indicator:
  (5) $$w_{j}=\frac{g_{j}}{\sum_{i}^{m}g_{j}}(0\leq w_{j}\leq 1,\sum_{j=1}^{n}w_{j}=1)$$

Based on relevant research, after calculating the weight of each index in different years, the comprehensive weight of secondary indicators is computed by $w_{j}^{*}=\overset{n}{\underset{j=1}{\sum }}w_{j}/n$ as the final weight of the evaluation index (as shown in the last column of Table 1).

(5) The comprehensive evaluation function is:

(6) $$U_{i}=\sum_{j=1}^{n}w_{ij}y_{ij}$$

where $U_{1}$, $U_{2}$ and $U_{3}$ are, respectively, the comprehensive evaluation scores of the tourism system, ecological environment system and public service system, and $w_{ij}$ is the weight. $\overset{n}{\underset{j=1}{\sum }}W_{ij}=1$ and $y_{ij}$ is the normalized value.

#### 3.3.2. Coupling Coordination Evaluation Model

The degree of coupling coordination is a measure of the mutual influence of two or more complex systems. The degree of coupling is a measure of the degree of interaction between systems, and the degree of coordination is a measure of the degree of mutual cooperation between systems [41]. The coupling degree function is used to reveal the internal synergy mechanism of interaction and mutual influence among tourism-ecological environment-public service in the basin. However, the coupling function can only describe the degree of coordinated development between subsystems and cannot determine whether the systems are closely related at a high level or mutually exclusive at a low level. Therefore, this paper further cites the CCD function to further explore the degree of coordinated development among the three, so as to reflect the level of coordinated development.

The coupling function formula is:

(7) $$C=\frac{3{\left(U_{1}\times U_{2}\times U_{3}\right)}^{\frac{1}{3}}}{U_{1}+U_{2}+U_{3}}$$

where, C is the coupling degree of tourism, the ecological environment and the public service system, and the value is range from 0 to 1. The larger the C value is, the higher the coupling degree will be. Conversely, the coupling degree will be lower. Based on the coupling degree, the comprehensive evaluation index T is used to construct the CCD model of tourism, ecological environment and public service system to further determine the coordinated development of the three systems.

Coupling coordination function:

(8) $$T=\alpha U_{1}+\beta U_{2}+\delta U_{3}$$

where the CCD D is introduced to represent the coupling coordination scheduling of each subsystem. The larger the value of D is, the higher the coupling coordination relationship of the subsystem, and vice versa, will be.

(9) $$D=\sqrt{C\times T}$$

where T is the comprehensive development index value of the public service system, and D is the coupled co scheduling of tourism, ecological environment and public service system. α,β and δ are undetermined coefficients, and they, respectively, represent the contribution of tourism, ecological environment and public service, whose weights are assigned by α=β=δ=1/3, respectively. The value of D is range from 0 to 1. The greater the D value is, the better the CCD, and vice versa, will be. At the same time, referring to the classification of CCD by existing scholars [42], the coordination degree of tourism ecological environment public service is divided into ten categories, as shown in Table 2.

#### 3.3.3. GM (1,1) Grey Prediction Method

Grey system theory is featured by small sample size, simple calculation, and high short-term prediction accuracy. Since grey system theory was proposed [43,44], it has been a mathematical method to solve the incomplete information system and issues without full information. According to the existing classification [45] and its application range [46], the forecasting model of the sequence is used in this paper to predict the evolution trend of tourism-ecological environment-public service in the Yellow River Basin. If there are m observations in the original data time series $x^{\left(0\right)}=\{x^{\left(0\right)}\left(1\right),x^{\left(0\right)}\left(2\right),x^{\left(0\right)}\left(3\right)\ldots x^{\left(0\right)}\left(m\right)\}$, the original time series data will be accumulated once to generate a new time series $x^{\left(1\right)}=\left\{x^{\left(1\right)}\left(1\right),x^{\left(1\right)}\left(2\right),x^{\left(1\right)}\left(3\right)\ldots x^{\left(1\right)}\left(m\right)\right\}$. Then, the first-order differential equation of the GM (1,1) model is:

(10) $$dx^{\left(1\right)}/dt+\alpha x^{\left(1\right)}=\mu$$

where α is the grey development coefficient; μ is the grey effect; *t* is the time. The standard solution in the GM (1,1) model is as follows:

(11) $${\hat{x}}^{\left(1\right)}s\left(t+1\right)=\left[x^{\left(0\right)}-\frac{\mu }{\alpha }\right]e^{\alpha t}+\frac{\mu }{\alpha }$$

The specified time series value can be predicted by this formula. In order to verify the reliability of the model, this paper draws on the accuracy test of the prediction results by existing scholars [47] to obtain the accuracy of the residuals (C) and ensure the rationality of the results (p), so as to check whether the modified model GM (1,1) is qualified. As shown in Table 3.

#### 3.3.4. PVAR Model

The advantage of the panel vector autoregression (PVAR) model is that it allows all variables to be endogenous and reflects the dynamic influence relationship of multiple variables. It not only takes into account the differences of each individual, but also effectively solves the endogeneity of variables by introducing variable lags in the model [48,49]. As this paper studies the interaction between tourism-ecological environment-public service, there may be a two-way mechanism, leading to endogeneity problems. Therefore, based on the analysis of CCD, this paper further explores the dynamic coupling relationship of tourism, the ecological, environment and public service system, and establishes a panel vector autoregressive (PVAR) model, which effectively avoids endogeneity and multicollinearity. Based on relevant research, the PVAR model established in this paper is as follows:

(12) $$Y_{it}=\theta_{0}+\sum_{j=1}^{k}\theta_{j}Y_{it-j}+\alpha_{i}+\beta_{i}+u_{it}$$

where $Y_{it}$ represents column vector of tourism, ecological environment and public service in each province. $\theta_{0}$ represents the intercept item, $\theta_{j}$ represents the lag *J*-order matrix, $\alpha_{i}$ indicates individual effects, $\beta_{i}$ represents the time effect, and $u_{it}$ represents random error.

## 4. The Static Relationship Analysis among Tourism-Ecological Environment-Public Service in the Yellow River Basin

### 4.1. Analysis of the Development of Each Subsystem of Tourism-Ecological Environment and Public Service in the Yellow River Basin

**Figure 1:**
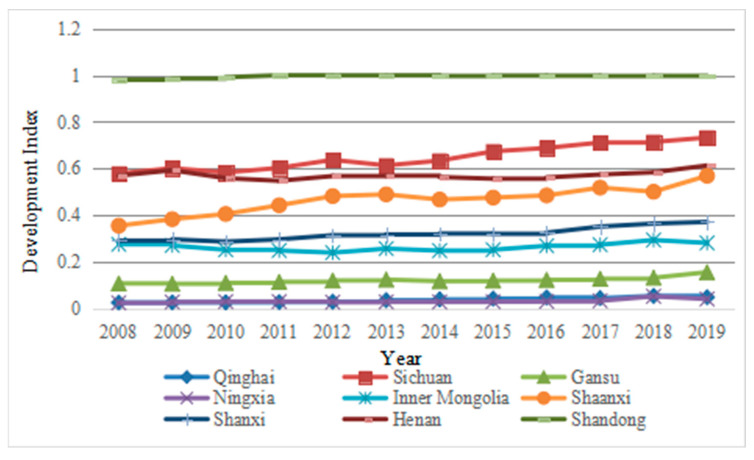
Development Status of Tourism in the Yellow River Basin from 2008 to 2019.

**Figure 2:**
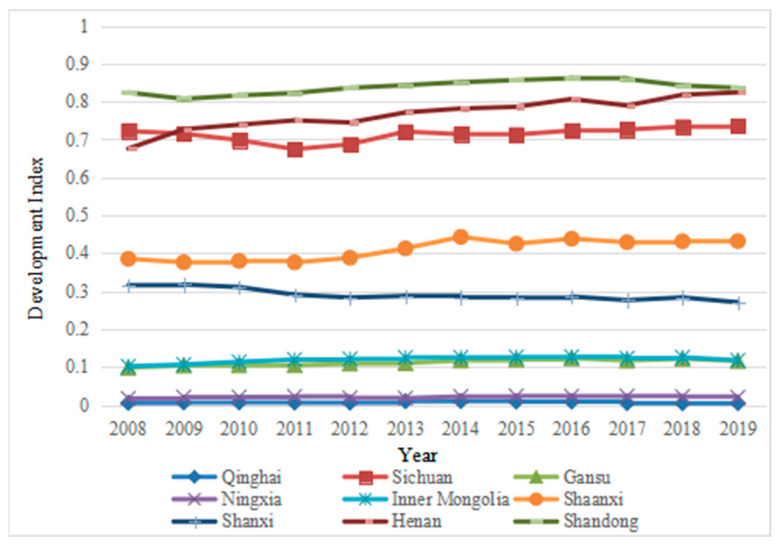
Development Status of Public Service in the Yellow River Basin from 2008 to 2019.

**Figure 3:**
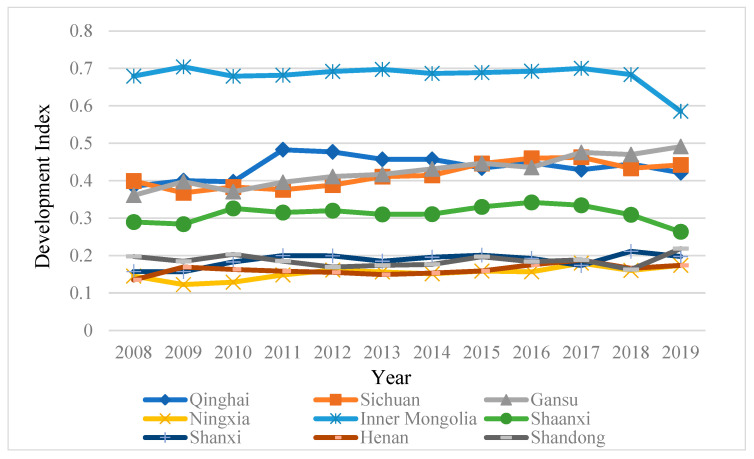
Ecological Environment Development Status of Nine Provinces in the Yellow River Basin from 2008 to 2019.

**Figure 4:**
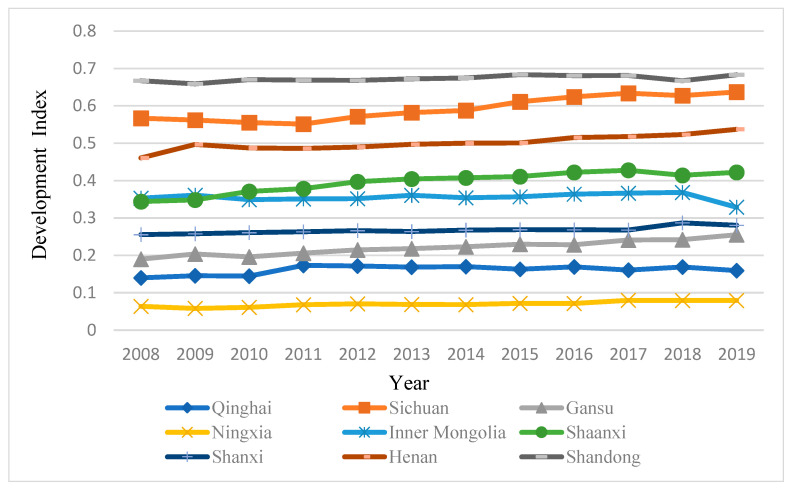
Comprehensive Development Status of Tourism, Ecological Environment, and public service in Nine Provinces of the Yellow River Basin from 2008 to 2019.

The comprehensive evaluation scores of each subsystem of tourism-ecological environment-public service in the Yellow River Basin from 2008 to 2019 are shown in Figure 1, Figure 2, Figure 3 and Figure 4.

#### 4.1.1. Analysis of the Development Status of Tourism-Public Service in the Yellow River Basin

It can be seen from Figure 1 and Figure 2 that there is a strong similarity in the development of tourism and public service systems in the Yellow River Basin. The top three are Shandong, Sichuan and Henan provinces, whose trends are roughly the same. After years of development, the tourism and public service systems of Shandong province have always been the number one. Tourism in Sichuan and Henan has been stable for many years, maintaining second or third place. However, the public service of Sichuan Province has dropped from second in 2008 to third in 2019, and the public service of Henan Province has risen from third in 2008 to second in 2019. In addition, Qinghai and Ningxia are at the end of the two systems, indicating that the development of the regional tourism industry and the level of public service are strongly correlated, and the development of the tourism industry will provide support for improving the quality of regional public service. Conversely, the lagging development of the tourism industry will restrict the quality of public service in the region. Compared with tourism and public service, the curve of the former fluctuates, and the curve of the latter is relatively flat, which shows that the public service in the Yellow River Basin will lead to long-term differences in infrastructure between regions. The imbalance of resource allocation is prominent, and it is difficult to improve the economic benefits in the short term. As the development of the tourism industry is dependent on the resource and environment industry, the development of the tourism industry in different regions of the river basin is different. Therefore, under the guidance of proper development and favorable policies, the development of the tourism industry will be cleaner and more efficient. It is of great significance to promote the coordinated development of the tourism belt in the Yellow River Basin.

#### 4.1.2. Analysis of the Development Status of the Ecological Environment in the Yellow River Basin

It can be seen from Figure 3 that the change characteristics of the ecological environment in the nine provinces of the Yellow River Basin are quite different from the tourism and public service systems. Inner Mongolia Autonomous Region, Gansu Province, and Sichuan Province ranked the top three in ecological environment evaluation values, while Ningxia Hui Autonomous Region, Shanxi Province and Henan Province ranked at the bottom, which shows that the ecological environment is not significantly related to tourism and public service, and there is no conflict between developing the tourism industry and protecting the ecological environment. Under the guidance of carbon peaking and carbon neutrality goals, it is important to advocate ecological protection and environmental optimization to protect the ecological environment when developing the regional tourism industry and improving infrastructure. The ecological environment evaluation of the nine provinces along the Yellow River Basin is primarily featured by rising volatility. The provinces with rising volatility include Gansu, Sichuan, Henan, Shandong, and Shanxi. The ecological environment in these areas has suffered greater damage. During the development process, the government implemented friendly policies, which effectively alleviated the deterioration of the regional ecological environment and promoted the balanced development of the three systems of tourism-ecological environment-public service in the Yellow River Basin. The provinces that increased first and then decreased include Inner Mongolia Autonomous Region, Qinghai Province, and Shaanxi Province, the ecological environment development of which conforms to the positive U-shaped environmental Kuznets curve. In the early stage of development, the regional structure was relatively optimized, tourism resources were properly developed, environmental protection was intensified, and the ecological environment coordination value was increased too. Due to the excessive pursuit of economic growth and the rapid development of the tourism industry, the ecological environment has deteriorated, and the ecological environment coordination value has also shown a fluctuating downward trend.

#### 4.1.3. Analysis of the Comprehensive Development Status of Tourism-Ecological Environment-Public Service in the Yellow River Basin

According to the coupling coordination function, the comprehensive evaluation index of the three systems of tourism-ecological environment-public service in the Yellow River Basin can be calculated to reflect the differences in the comprehensive development of tourism-ecological environment-public service in the nine provinces of the Yellow River Basin. As shown in Figure 4, according to the change trend of the comprehensive evaluation index of the three systems in the Yellow River Basin from 2008 to 2019, the nine provinces along the route can be divided into four categories. The first category (0.60–0.70) is Sichuan and Shandong, and the comprehensive evaluation index of Shandong has been in the leading position from 2008 to 2019. The second category (0.4–0.6) is Henan and Shaanxi, the comprehensive development index of which showed a fluctuating upward trend; the third category (0.2–0.4) is Gansu, Inner Mongolia and Shanxi, relying on the advantages of local coal and natural gas resource to promote regional industrialization so that the public service infrastructure in the region has been improved. The fourth category (0–0.20) is Qinghai and Ningxia Hui Autonomous Regions. To sum up, except for Sichuan Province, the comprehensive development level of tourism-ecological environment-public service in the Yellow River Basin is getting higher from the west to east in spatial distribution.

### 4.2. Spatial and Temporal Analysis of the Coupling Coordination of Tourism-Ecological Environment-Public Service in the Yellow River Basin

The CCD index of tourism-ecological environment-public service in the Yellow River Basin can be obtained by substituting the comprehensive evaluation values of tourism-ecological environment-public service in the nine provinces along the Yellow River Basin into the coupling degree function and coupling coordination function formula. As shown in Table 4. From the perspective of time evolution, the changes in the CCD of tourism-ecological environment-public service in the nine provinces along the Yellow River Basin from 2008 to 2019 maintained a stable upward trend and generally headed for harmonious and high-quality development. Among them, the CCD levels of Sichuan, Henan, Shandong, Inner Mongolia, Ningxia, and Qinghai remained unchanged during the sample period, indicating that the coordinated development relationship between tourism-ecological environment-public service in these provinces is relatively stable. The CCD level of Gansu, Shaanxi, and Shanxi rose to the first level coupling range during the sample period. The coupling coordination level of Gansu and Shanxi rose from a slight imbalance to near misalignment, and the coupling coordination level of Shaanxi rose from barely coordination to primary coordination. In general, based on the actual situation, the provinces along the Yellow River need to take further measures to innovate the development model of the tourism industry, optimize the quality of the ecological environment and improve the construction of public service infrastructure, so as to improve the coordination of tourism-ecological environment-public service in the Yellow River Basin and prevent regional economic development from being imbalanced.

In order to compare the coupling and coordinated development levels of the three systems horizontally in the nine provinces along the Yellow River Basin, the mean values of the coupling coordination index of tourism-ecological environment-public service in the Yellow River Basin from 2008 to 2019 are listed in Table 5. The CCD of tourism-ecological environment-public service in the Yellow River Basin is spatially similar to the distribution of the comprehensive evaluation indices of the three major systems, which is generally low in the west and high in the east except for Sichuan province. In the lower areas of the Yellower River, Shandong province reaches intermediate coordination. Then, Henan and Shaanxi, in the middle reaches of the Yellow River, reach the primary coordination, and Shanxi reached barely coordination. In the upper reaches of the Yellow River, Qinghai and Ningxia are a moderate imbalance, Gansu is near misalignment, and Inner Mongolia is barely disordered. The main reason is that compared with the western region, the tourism system and public service system in the eastern region have significant advantages, which compensate for the imbalance coefficient to a certain extent. In addition, combined with the evaluation indexes of subsystems in the nine provinces of the Yellow River Basin, it can be seen that the main factors restricting the level of coupling coordination are also different. The four provinces and Sichuan province, in the middle and lower reaches of the Yellow River Basin, belong to a lagged type of ecological environment, and the destruction of the ecological environment system is the main reason that restricts the coordinated development of these regions; while provinces in the upper reaches of the Yellow River Basin, such as Qinghai, Inner Mongolia, Gansu and Ningxia autonomous regions, belong to a lagged type of public service.

### 4.3. Forecast of Coordination Development of Tourism-Ecological Environment-Public Service Coupling in the Yellow River Basin

Based on the GM (1,1) prediction model, this paper predicts and analyzes the CCD of the three subsystems in the nine provinces of the Yellow River Basin from 2008 to 2019. The results are shown in Table 6. It can be seen from the table that in the next five years, the variation characteristics of the CCD of tourism-ecological environment-public service in the Yellow River Basin will continue the features from 2008 to 2019 and maintain an upward trend. Except for Shandong province, the degree of coupling and coordination decreased, and eight other provinces showed a slight upward trend. Among them, Sichuan province has risen from intermediate coordination to good coordination, while the CCD of the other seven provinces has increased, but they still maintained the original coordination level. The forecast results show that the CCD of tourism-ecological environment-public service in most provinces in the Yellow River Basin will be improved in the next five years, but the level of coupling and the speed of coordinated development are still slow. Sichuan is the only province, the three systems of which reach a state of mutual promotion and coordination. However, it still takes a long time for other provinces to do that. Therefore, in the future, it is required that Sichuan province should be the core of the upstream and Shandong and Henan Provinces should be the core of the downstream, so as to build a double-core circle in the Yellow River Basin and radiate to other provinces and regions in the whole basin, strengthen the interconnection between various regions, make up for their own shortcomings, and realize the coordinated development of tourism-ecological environment-public service in the whole basin.

### 4.4. The Dynamic Relationship Analysis among Tourism, Ecological Environment, and Public Service

In order to further analyze the dynamic relationship between tourism, ecological environment and public service, we use the PVAR model to further analyze the relationship between the comprehensive index values of the three systems in nine provinces along the Yellow River Basin in China from 2008 to 2019. The following is a stationarity test according to the steps of PVAR model establishment, which confirms the robustness and accuracy of the data analysis results.

#### 4.4.1. Unit Root Test

In order to make the autoregressive results authentic and reliable, the unit root test was first conducted on the comprehensive index values of the three systems of tourism (TE), ecological environment (EE) and public service (TPS) by adopting six methods: i.e., LLC test, IPS test, ADF test, HT test, Breitung test and LM Test. The test results are shown in Table 7.

It can be seen from Table 7 that the original sequence of TE, EE and TPS variables is unstable, and the last three first-order differences have passed the 5% significance level test. That is to say, all variables are stable after the first order differential processing.

#### 4.4.2. Cointegration Test

The premise of the cointegration test is the same order document. Since the three variables in this paper tend to be stable after first-order differential processing, the Kao test and Pedroni test are selected. The *p* values of the test results are less than 0.05, as shown in Table 8, indicating that there is a stable and balanced relationship between tourism, ecological environment and public service.

#### 4.4.3. Optimal Lag Order and Granger Causality Test

The selection criteria of AIC, BIC and HQIC minimum values are used in this paper, and the optimal lag order is 1, as shown in Table 9.

Granger causality is used to test the causal relationship between tourism-ecological environment-public service in nine provinces in the Yellow River Basin. The results are shown in Table 10. During the sample period, the Granger causality of the comprehensive evaluation index of the ecological environment is not the comprehensive evaluation index for tourism and the Granger causality of the comprehensive evaluation index for public service is not the comprehensive evaluation index for the ecological environment, which were accepted, but the null hypothesis was rejected. Therefore, this indicates that there is a two-way Granger causality between tourism and public service. That is to say, the development of regional tourism will improve the quality of regional public service, and at the same time, the improvement of the quality of regional public service will have an impact on tourism development.

#### 4.4.4. Impulse Response

**Figure 5:**
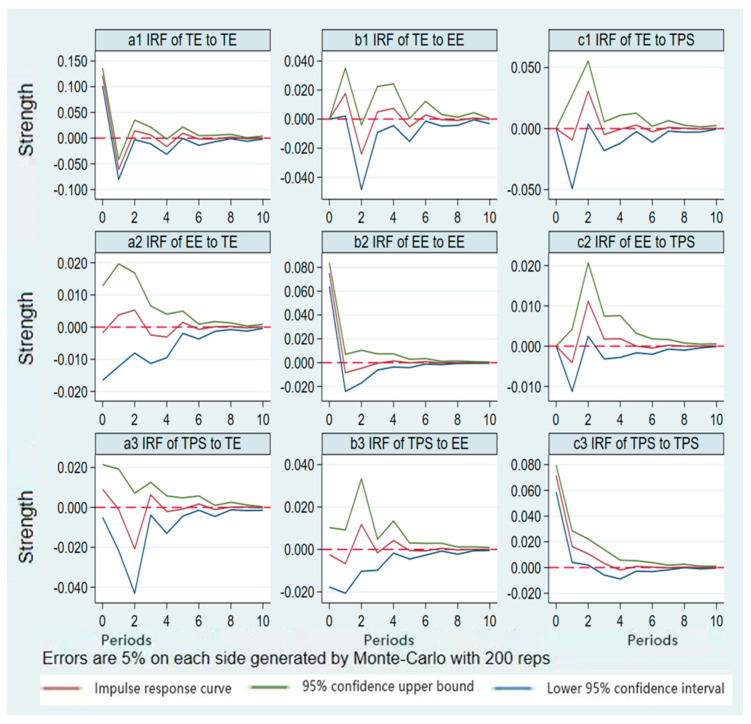
Impulse Response Diagram of Tourism-Ecological Environment-Public-Service.

In this paper, the impulse response analysis is used to intuitively observe the dynamic relationship between tourism, the ecological environment and public service. One standard deviation shock was given to tourism, the responses of the ecological environment and public service in periods from 0 to 10 can be obtained by using the Monte Carlo method to simulate 500 times. The impulse responses and cumulative impulse response trajectories of the dynamic impact of tourism on the ecological environment and public service are shown in Figure 5. The horizontal axis represents the number of impulse response periods (in years), the maximum lag period of which is 10, and the vertical axis is the response degree of the variable to the shock.

Figure 5(a1) shows that tourism has a standard deviation impact on itself. Tourism shows a positive response in the current period, a negative response in the first period, a positive response in the second period, and then gradually decreases until it is stable; Figure 5(b1) shows that tourism has no response to a standard deviation impact on the ecological environment in the current period. The first period produces a positive response, the second period decreases and produces a negative response, and the fifth period slowly converges to zero. Figure 5(c1) shows that tourism has no response to a standard deviation impact on public service in the current period. In the first period, it shows a negative response. In the second period, it shows a positive impact and reaches its peak, then gradually decreases and tends to 0. This shows that tourism has a positive lagged effect on the ecological environment and a negative lagged effect on public service.

Figure 5(a2) shows that the ecological environment has a standard deviation impact on tourism. Tourism shows a negative response in the current period, which shows a positive response and reaches a peak in the first period. In the third period, tourism shows a declining and negative response, and then gradually declines and slowly converges to 0; Figure 5(b2) shows that the ecological environment has a standard deviation impact on itself, the ecological environment has a positive response in the current period, and the first period shows a negative response and slowly converges to 0; Figure 5(c2) shows that the ecological environment has no response to a standard deviation impact on public service. The first period shows a negative response. The second period shows a positive impact and reaches a peak, and then gradually declines and tends to be stable. This shows that the ecological environment has a negative lagging effect on tourism and public service. If the quality of the ecological environment continues to deteriorate, tourism development will be hindered and the quality of the ecological environment will be worse.

Figure 5(a3) shows that the public service has a standard deviation impact on tourism. Public service shows a positive response in the current period and shows a positive response in the first period. In the second period, it declines and reaches the minimum value. In the third period, it rises and shows a positive response, then gradually decreased, and slowly converged to 0; Figure 5(b3) shows that public service has a standard deviation impact on the ecological environment. Public service shows a negative response in the current period, and a positive response in the second period, then it begins to gradually decrease and slowly converges to 0. Figure 5(c3) shows that public service shows a positive response to a standard deviation impact on itself in the current period, which shows a positive response in the first period, and then gradually decreases and tends to be stable. This shows that public service has a positive lagging effect on tourism and the ecological environment. If the public service in the region is improved, the development level of the tourism industry and the quality of the ecological environment in the latter periods will be improved too.

#### 4.4.5. Variance Decomposition Analysis

To show the degree of mutual influence among tourism, the ecological environment and public service development more accurately, variance decomposition is used to further evaluate the importance of the effects of various shocks. The method of predicting errors of endogenous variables can be decomposed into endogenous variables and related parts according to their causes by variance decomposition, so as to explain the explanatory power of each variable to endogenous variables.

In this paper, variance decomposition results under the 95% confidence interval are calculated by running 500 Monte Carlo simulations on variables. The results are shown in Table 11, which tend to be stable in the sixth period. In the tourism variance decomposition, the first period itself contributed 100% of the explanatory power. The ecological environment and public service contributed 4.6% and 5% of the explanatory power in the fifth period, which changed to 4.8% and 5.0%, respectively, in the tenth period. The inter-temporal comparison shows that tourism mainly relies on the relationship of its own, and the contribution of the ecological environment and public service is on the rise. In the decomposition of ecological environment variance, public service contributes 0% in the first period, and rises to 2.5% in both the fifth and tenth periods and tends to be stable, which means that improving the public service infrastructure will help promote the long-term improvement of the ecological environment. The method contribution of tourism to the ecological environment contributes 0.1% in the first period and rises to 1.1% in the sixth period and tends to be stable, which means that the driving force of tourism to improve the quality of the ecological environment needs to be improved. In the variance decomposition of public service, the contribution of tourism to public service increases slowly from 1.6% in the first period to 9% in the tenth period, and the contribution of the ecological environment to public service increases slowly from 0.1% in the first period to 3.4% in the tenth period, which means that the driving force of tourism and ecological environment to improve the public service base needs to be strengthened.

## 5. Discussion and Policy Recommendations

### 5.1. Discussion

The CCD of tourism and ecological environment system in the Yellow River Basin in this paper is the same as the existing research results [50]. The coordination degree of most provinces is on the rise, and the overall level of tourism coupling coordination in the lower reaches of the Yellow River is higher than that in the upper reaches. Some scholars [51] have explored the interactive response analysis results of tourism ecological efficiency and tourism economic development level in the Yellow River Basin. The impulse responses of tourism and the ecological environment show a smooth response trend, with large fluctuations in the early stage and stability in the later stage. The degree of mutual contribution between regional tourism and the ecological environment has gradually increased over time, especially in Henan and Sichuan provinces. However, the coordination degree of tourism and ecological environment in this paper is inconsistent with the high coordination of existing research results, and the coordination degree of Ningxia remains unchanged. Tourism plays a significant role in promoting the ecological environment and public service.

### 5.2. Policy Recommendations

(1) Pursue win-win cooperation and promote regional coordination. The development of the nine provinces of the Yellow River is diversified. On the one hand, regional opening and cooperation should be actively promoted to break down regional administrative barriers and build an integrated development mechanism to achieve market co-construction, brand development, and benefit sharing. On the other hand, it is necessary to insist on a new direction of tourism development in the Yellow River Basin and condense the cultural brand effect of the Yellow River Basin, so as to achieve coordinated development of tourism, ecological environment and public service with complementary advantages in the upper, middle and lower reaches of the Yellow River Basin.

(2) Pursue open development and innovative talent cultivating models. We should strengthen the support of talents, open research, and expand the influence. We should introduce high-level talents and scarce talents in short supply in the field of cultural tourism, establish a mechanism for joint training of talents by professional colleges, scientific research institutes, and training institutions, and build tourism think tanks, professional talent information bases, and online talent markets. We should actively cultivate research teams and expand the team of Yellow River researchers. It is recommended to strengthen horizontal integration, promote the interdisciplinary integration of disciplines, and regularly organize academic seminars on the theme of “Tourism in Yellow River Basin” in the existing academic framework system, so as to expand the influence of research in this field.

(3) Formulate differentiated tourism policies according to local conditions. The improvement of the policy system is the guarantee for the effective development of the Yellow River Basin. It is necessary to pay attention to the overall planning and coordination of policies in this field and formulate differentiated regional development strategies according to the coordination degree of tourism, ecological environment and public service in the nine provinces of the Yellow River Basin and in combination with the social and economic development conditions and cultural conditions of the region. For provinces with low coupling coordination among the three systems, namely, Qinghai, Ningxia, Gansu, and Inner Mongolia, government support should be strengthened and the current situation of low utilization of tourism resources should be improved by policy and financial support; for provinces with moderate coupling coordination among the three systems, Shaanxi and Shanxi actively carry out linkages with tourism resources in surrounding cities, improve the development of tourism industry in the region to achieve regional coordinated development through industrial agglomeration; for provinces with high coupling and coordination of the three systems, Sichuan, Henan and Shandong, comprehensive governance should be strengthened to standardize cultural tourism market order and upgrade the structure of tourism and the tertiary industry.

## 6. Conclusions

The coupling mechanism of the three systems of tourism, ecological environment and public service is analyzed in this paper. Based on this, a comprehensive index system of the three systems is constructed. The comprehensive level and CCD of the tourism, ecological environment and public service are calculated by using the data of nine provinces in the Yellow River Basin from 2008 to 2019, and the relationship among the three is empirically analyzed. The conclusions are as follows.

First, tourism and public service in the nine provinces of the Yellow River Basin are strongly correlated. The top three of the two systems are Shandong, Sichuan and Henan, and they show the same changing trends, while Qinghai and Ningxia are at the end. The two systems of Shaanxi, Shanxi, Inner Mongolia and Gansu are volatile, which means that under the guidance of proper development and favorable policies, and the improvement of supporting infrastructure, the tourism industry can promote the coordinated development of tourism and public service and narrow the gap in regional industrial development. The ecological environment system is dominated by rising volatility, which means that ecological protection and environmental friendliness are not in conflict with tourism development, and regions can adopt various measures to jointly promote the coordinated development of the three.

Second, the CCD of the tourism-ecological environment-public service system in the Yellow River Basin maintained a steady increase from 2008 to 2019. Among them, the CCD levels of the six provinces of Sichuan, Henan, Shandong, Inner Mongolia, Ningxia Autonomous Region, and Qinghai remained unchanged during the sample period, and the CCD level of the three provinces of Gansu, Shaanxi and Shanxi rose to the upper-level coupling range during the sample period. From a spatial point of view, the CCD of the three systems of tourism-ecological environment-public service in the Yellow River Basin presents a spatial pattern of low upstream and high downstream. At the same time, the main factors restricting the coordinated development of different provinces are also different. Sichuan, Henan, Shandong, Shaanxi and Shanxi belong to the lag type of ecological environment, and Inner Mongolia, Qinghai, Gansu and Ningxia belong to the lag type of public service. For different types of development lags in different regions, local governments should implement differentiated policies, make full use of their own advantages, make up for their own shortcomings, and promote the coordinated development of regional industries.

Third, the CCD of the tourism-ecological environment-public service system in the Yellow River Basin will continue to maintain a steady upward trend in the expected five years. Except for Shandong Province, which has a certain degree of decline, the rest of the eight provinces and regions have shown a slight increase trend. Among them, Sichuan Province has risen from intermediate coordination to good coordination, while the CCD of the other seven provinces has increased but still maintains the original coordination level. In general, the degree of coupling and coordination of the provinces has been rising slowly with a low level of development, and the development of various regions is still uneven. Thus, it is necessary to strengthen the mutual connection among regions and make up for their own shortcomings, so as to achieve the coordinated development of tourism, ecological environment and public service in the whole basin.

Fourth, the three systems of tourism, ecological environment and public service in the Yellow River Basin interact with each other. Public service plays a significant role in promoting tourism, and tourism can also significantly promote the ecological environment and public service, indicating the development of tourism and public service in the Yellow River Basin. There is a positive mutual promotion between them. It shows that the development of the tourism industry in the Yellow River Basin can not only improve the environment of the region, but also improve the quality of public service in the region. At the same time, the improvement of public service quality and eco-friendly development also counteract the development of the tourism industry and help the regional government to connect the tourism industry chain upstream and downstream.

However, we have to admit that there are some limitations to the paper. To be specific, the data selected in this paper are from provinces, which cannot truly reflect the real situation of the three systems in the Yellow River Basin, and there are certain errors in the research results. Thus, urban data or county data will be considered first for future research conclusions. Secondly, due to the randomness of the original data, though the grey prediction model is adopted, errors may be made in the results. Finally, the discussion of this paper is mainly based on the three systems without a deep analysis of the influencing factors. Therefore, the coordinated development and internal drive of tourism-ecological environment-public service in the Yellow River Basin will be the research direction in the future.

## Figures and Tables

**Table 1:** The evaluation index system on Tourism, Ecological Environment and Public Service in the Yellow River Basin.

Subsystem	Primary Index	Weight	Secondary Index	Unit		Comprehensive Weight
Tourism system	Tourism scale	0.574	Number of inbound tourists	Ten thousand times	+	0.117
			Number of domestic tourists	Ten thousand times	+	0.112
			Tourism practitioners	people	+	0.119
			Number of star hotels	individual	+	0.146
			Number of travel agencies	individual	+	0.080
	Tourism benefits	0.426	International tourism revenue	Million dollars	+	0.141
			Domestic tourism revenue	RMB100 mn	+	0.107
			Per capita GDP	element	+	0.054
			Total retail sales of social consumption	RMB100 mn	+	0.125
Ecological environment system	Tourism environment	0.216	Total energy consumption	10,000 tons	−	0.034
			Green space rate of built-up area	%	+	0.050
			Sewage treatment rate	%	+	0.033
			Carbon dioxide emissions	10,000 tons	−	0.037
			Harmless treatment rate of domestic waste	%	+	0.030
			Total sewage discharge	Ten thousand cubic meters	−	0.032
	Tourism ecology	0.784	Wetland area	10,000 hectares	+	0.147
			forest coverage	10,000 hectares	+	0.106
			Number of nature reserves	individual	+	0.259
			Area of Nature Reserve	hectare	+	0.185
			afforestation area	hectares	+	0.088
public service system	Urban infrastructure	0.594	Number of medical institutions	individual	+	0.149
			Number of beds	Zhang	+	0.162
			Number of health technicians	people	+	0.140
			Number of public toilets	individual	+	0.143
	Scientific research education	0.406	Number of colleges and Universities	place	+	0.095
			College graduates	people	+	0.116
			Number of people engaged in scientific and technological activities	people	+	0.196

Note: ‘+’ indicates positive indicators and ‘−’ indicates negative indicators.

**Table 2:** The Classification of CCD.

Coupled co scheduling	[0.0, 0.1)	[0.1, 0.2)	[0.2, 0.3)	[0.3, 0.4)	[0.5, 0.6)
Coordination level	I extreme disorder	II severe disorder	III moderate disorder	IV mild disorder	V near maladjustment
Coupled co scheduling	[0.5, 0.6)	[0.6, 0.7)	[0.7, 0.8)	[0.8, 0.9)	[0.9, 1.0)
Coordination level	VI coordination	VII Primary coordination	VIII intermediate coordination	IX good coordination	X high quality coordination

**Table 3:** Grade Standard of Grey Prediction Accuracy Test.

Accuracy Class	P	C	Accuracy Class	P	C
good	>0.95	<0.35	good	>0.70	<0.65
qualified	>0.80	<0.50	qualified	≤0.70	≥0.65

**Table 4:** Time Evolution of Tourism, Ecological Environment and Public Service CCD in the Yellow River Basin from 2008 to 2019.

	2008	2009	2010	2011	2012	2013	2014	2015	2016	2017	2018	2019
Qinghai	0.200	0.208	0.209	0.214	0.215	0.237	0.242	0.238	0.243	0.217	0.222	0.219
Sichuan	0.742	0.736	0.734	0.731	0.745	0.753	0.757	0.774	0.782	0.788	0.781	0.788
Gansu	0.397	0.407	0.405	0.412	0.421	0.424	0.428	0.433	0.435	0.440	0.445	0.457
Ningxia	0.203	0.208	0.212	0.219	0.213	0.212	0.218	0.223	0.223	0.232	0.242	0.232
Inner Mongolia	0.518	0.523	0.520	0.523	0.523	0.532	0.528	0.530	0.537	0.537	0.543	0.519
Shaanxi	0.585	0.588	0.608	0.612	0.626	0.630	0.633	0.637	0.646	0.649	0.637	0.634
Shanxi	0.494	0.496	0.504	0.509	0.511	0.507	0.512	0.513	0.512	0.507	0.529	0.521
Henan	0.611	0.647	0.638	0.634	0.635	0.635	0.638	0.641	0.656	0.664	0.656	0.667
Shandong	0.736	0.726	0.740	0.731	0.722	0.726	0.728	0.743	0.735	0.738	0.718	0.753

**Table 5:** Comparison of Mean Values of Tourism, Ecological Environment and Public Service Coupling Coordination Among provinces in the Yellow River Basin from 2008 to 2019.

	Tourism	Ecosystem	Public Service	Coupling	Comprehensive Evaluation Value	Coupling Coordination	Type of Coordination	Main Constraints
Qinghai	0.040	0.436	0.007	0.307	0.161	0.222	Moderately disordered	Lagged public service
Sichuan	0.648	0.415	0.714	0.973	0.593	0.759	Intermediate Coordinator	lagged eco-environment
Gansu	0.124	0.425	0.113	0.821	0.221	0.425	On the verge of dysregulation	Lagged public service
Ningxia	0.035	0.153	0.022	0.694	0.070	0.220	Moderately disordered	Lagged public service
Inner Mongolia	0.266	0.681	0.120	0.784	0.355	0.528	Barely coordinated	Lagged public service
Shaanxi	0.466	0.311	0.410	0.985	0.396	0.624	Primary coordination	Lagged eco-environment
Shanxi	0.323	0.188	0.291	0.972	0.267	0.510	Barely coordinated	Lagged eco-environment
Henan	0.573	0.162	0.768	0.827	0.501	0.644	Primary coordination	Lagged eco-environment
Shandong	0.994	0.187	0.838	0.798	0.673	0.733	Intermediate Coordinator	Lagged eco-environment

**Table 6:** Prediction of coordinated development of tourism-ecological environment-public service coupling in provinces along the Yellow River Basin.

	2020	2021	2022	2023	2024
Qinghai	0.234	0.235	0.237	0.238	0.240
Sichuan	0.800	0.807	0.814	0.821	0.828
Gansu	0.457	0.463	0.468	0.473	0.479
Ningxia	0.239	0.242	0.245	0.249	0.252
Inner Mongolia	0.536	0.537	0.539	0.540	0.541
Shaanxi	0.655	0.660	0.665	0.670	0.675
Shanxi	0.523	0.526	0.528	0.530	0.532
Henan	0.664	0.666	0.669	0.672	0.675
Shandong	0.739	0.740	0.741	0.742	0.743

**Table 7:** Unit Root Test Results.

	LLC	IPS	ADF	HT	Breitung	LM	
TE	−1.147 (0.125)	−0.930 (0.176)	−2.897 ** (0.003)	−4.624 (0.000)	−0.873 (0.192)	3.416 *** (0.000)	First order stability
DTE	−3.997 *** (0.000)	−4.619 *** (0.000)	−6.147 *** (0.000)	−13.557 *** (0.000)	−5.841 *** (0.000)	3.408 *** (0.000)	
EE	−0.787 (0.216)	−0.640 (0.261)	−4.014 *** (0.000)	0.562 ** (0.004)	−0.868 (0.193)	3.244 *** (0.000)	First order stability
DEE	−1.440 ** (0.075)	−3.815 *** (0.000)	5.197 *** (0.000)	−0.320 *** (0.000)	−3.013 ** (0.001)	3.761 *** (0.000)	
TPS	−3.795 *** (0.000)	−0.810 (0.209)	−4.778 *** (0.000)	0.727 (0.293)	0.253 (0.600)	4.032 *** (0.000)	First order stability
DTPS	−1.938 ** (0.026)	−3.430 *** (0.000)	−4.771 *** (0.000)	−0.056 *** (0.000)	−3.057 ** (0.001)	2.120 ** (0.017)	

Note: ** *p* < 0.05, *** *p* < 0.01.

**Table 8:** Kao Test and Pedroni Test Results.

		Statistic	*p*-Value
Kao test	Modified Dickey-Fuller	2.017	0.022
	Dickey-Fuller	1.829	0.034
	Augmented Dickey-Fulle	2.860	0.002
	Unadjusted modified Dickey-Fuller	−4.112	0.000
Pedroni inspection	Unadjusted Dickey-Fuller	−3.284	0.001
	Modified Phillips-Perron	1.647	0.050
	Phillips-Perron	−1.729	0.042

**Table 9:** Optimal lag order.

Lag	AIC	BIC	HQIC
1	−5.121 *	−4.057 *	−4.694 *
2	−4.900	−3.477	−4.333
3	−4.376	−2.539	−3.653
4	−3.478	−1.158	−2.583
5	−2.745	0.146	−1.667

Note: * *p* < 0.1.

**Table 10:** Granger Causality Test Results.

Null Hypothesis	F Statistic	*p*-Value	In Conclusion
TE is not Granger causality for EE	6.496	0.039	reject
TE is not Granger causality for TPS	4.609	0.100	reject
EE is not Granger causality for TE	0.437	0.804	accept
EE is not Granger causality for TPS	7.473	0.024	reject
TPS is not Granger causality for TE	5.439	0.066	reject
TPS is not Granger causality for EE	2.673	0.263	accept

**Table 11:** Analysis of Variance Results.

Period	Shock Variable TE			Shock Variable EE			Shock Variable TPS		
	TE	EE	TPS	TE	EE	TPS	TE	EE	TPS
1	1	0	0	0.001	0.999	0	0.016	0.001	0.984
2	0.979	0.016	0.005	0.003	0.994	0.003	0.015	0.009	0.976
3	0.907	0.043	0.050	0.008	0.968	0.024	0.083	0.031	0.886
4	0.905	0.044	0.051	0.009	0.967	0.024	0.089	0.031	0.880
5	0.904	0.046	0.050	0.010	0.965	0.025	0.089	0.034	0.877
6	0.903	0.047	0.050	0.011	0.965	0.025	0.089	0.034	0.877
7	0.902	0.048	0.050	0.011	0.964	0.025	0.090	0.034	0.877
8	0.902	0.048	0.050	0.011	0.964	0.025	0.090	0.034	0.877
9	0.902	0.048	0.050	0.011	0.964	0.025	0.090	0.034	0.877
10	0.902	0.048	0.050	0.011	0.964	0.025	0.090	0.034	0.877

## Data Availability

Not applicable.
